# 2-Meth­oxy-3-(tri­methyl­sil­yl)phenyl­boronic acid

**DOI:** 10.1107/S1600536813031656

**Published:** 2013-11-23

**Authors:** Krzysztof Durka, Sergiusz Luliński, Janusz Serwatowski

**Affiliations:** a Physical Chemistry Department, Faculty of Chemistry, Warsaw University of Technology, Noakowskiego 3, 00-664 Warsaw, Poland

## Abstract

The mol­ecular structure of the title compound, C_10_H_17_BO_3_Si, features an intra­molecular O—H⋯O hydrogen bond; the boronic group group has an *exo*–*endo* conformation. In the crystal, the mol­ecules inter­act with each other by O—H⋯O hydrogen bonds, producing centrosymmetric dimers that are linked by weak π–π stacking inter­actions featuring specific short B⋯C contacts [*e.g*. 3.372 (2) Å], forming an infinite columnar structure aligned along the *a-*axis direction.

## Related literature

For structures of related *ortho*-alk­oxy aryl­boronic acids, see: Cyrański *et al.* (2012 ▶). For binding energies of other boronic acid dimers, see: Cyrański *et al.* (2008 ▶); Durka *et al.* (2012 ▶). For the *PIXEL* program, see Gavezzotti (2003 ▶). For the synthesis, see: Durka *et al.* (2010 ▶).
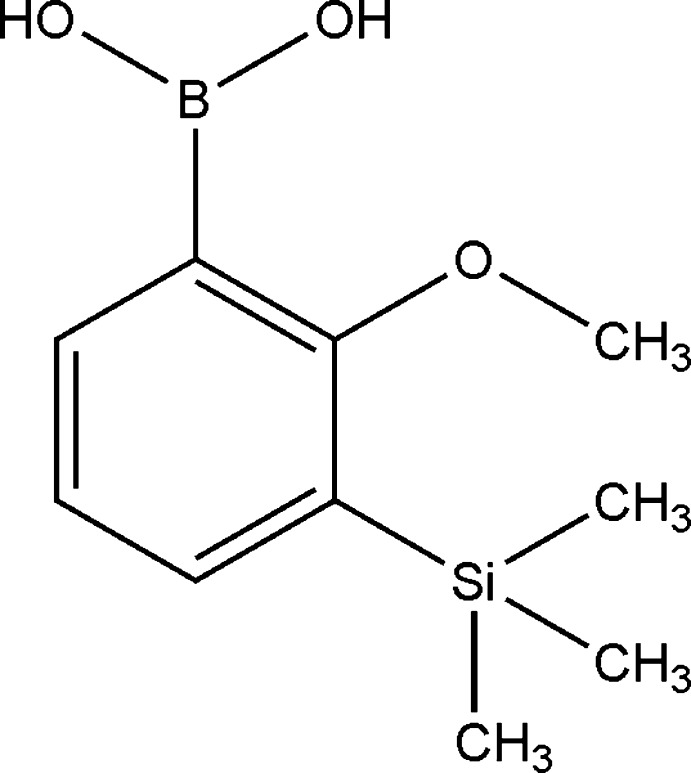


## Experimental

### 

#### Crystal data

C_10_H_17_BO_3_Si
*M* *_r_* = 224.14
Monoclinic, 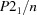

*a* = 9.1832 (11) Å
*b* = 9.7082 (10) Å
*c* = 14.1415 (16) Å
β = 104.26 (1)°
*V* = 1221.9 (2) Å^3^
*Z* = 4
Mo *K*α radiation
μ = 0.18 mm^−1^
*T* = 100 K
0.16 × 0.12 × 0.10 mm

#### Data collection

Bruker APEXII diffractometer
Absorption correction: multi-scan (Blessing, 1995 ▶) *T*_min_ = 0.744, *T*_max_ = 0.780
11175 measured reflections
2939 independent reflections
2154 reflections with *I* > 2σ(*I*)
*R*_int_ = 0.029

#### Refinement

*R*[*F* ^2^ > 2σ(*F* ^2^)] = 0.034
*wR*(*F* ^2^) = 0.097
*S* = 1.02
2939 reflections
136 parameters
1 restraint
H-atom parameters constrained
Δρ_max_ = 0.36 e Å^−3^
Δρ_min_ = −0.30 e Å^−3^

### 

Data collection: *APEX2* (Bruker, 2010 ▶); cell refinement: *SAINT* (Bruker, 2010 ▶); data reduction: *SAINT* and *SORTAV* (Blessing, 1995 ▶); program(s) used to solve structure: *SHELXS97* (Sheldrick, 2008 ▶); program(s) used to refine structure: *SHELXL97* (Sheldrick, 2008 ▶); molecular graphics: *DIAMOND* (Brandenburg, 2005 ▶); software used to prepare material for publication: *PLATON* (Spek, 2009 ▶).

## Supplementary Material

Crystal structure: contains datablock(s) global, I. DOI: 10.1107/S1600536813031656/tk5273sup1.cif

Structure factors: contains datablock(s) I. DOI: 10.1107/S1600536813031656/tk5273Isup2.hkl

Supplementary material file. DOI: 10.1107/S1600536813031656/tk5273Isup3.cml

Additional supplementary materials:  crystallographic information; 3D view; checkCIF report

## Figures and Tables

**Table 1:** Hydrogen-bond geometry (Å, °)

*D*—H⋯*A*	*D*—H	H⋯*A*	*D*⋯*A*	*D*—H⋯*A*
O2—H2⋯O1	0.84	2.04	2.7532 (14)	142
O3—H3⋯O2^i^	0.84	1.97	2.8051 (14)	175

Symmetry code: (i) 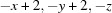.

**Fig. 1.**
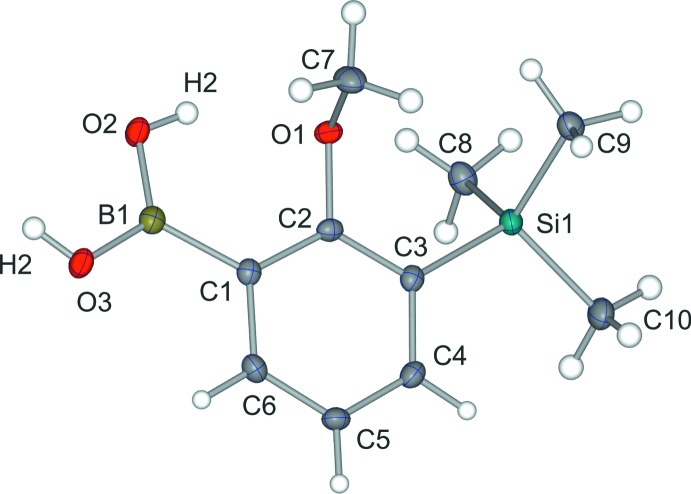
View of the title compound (I) with the atom numbering scheme. Displacement ellipsoids for non-H atoms are drawn at the 50% probability level.

**Fig. 2.**
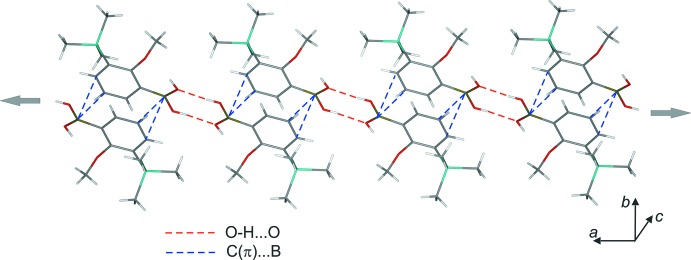
Part of the crystal structure of (I) showing formation of a column along the [100] direction. The O–H···O, C–H···O and C(π)···B interactions are depicted as red and blue dashed lines, respectively.

**Fig. 3.**
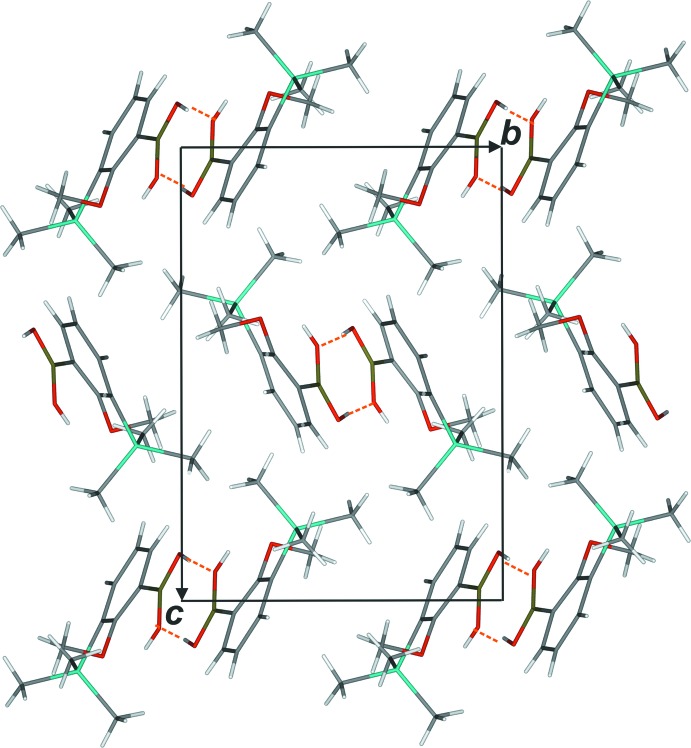
Packing diagram viewed along the a axis, indicating the columns of O—H···O and C(π)···B interacting molecules of (I).

## supplementary crystallographic information

### 2.1. Synthesis and crystallization

The preparation of the title compound (I) was described previously (Durka *et al.*, 2010). Crystals suitable for single-crystal X-ray diffraction analysis were grown by slow evaporation of an acetone solution of (I).

### 2.2. Refinement

Crystal data, data collection and structure refinement details are summarized in Table 1. All hydrogen atoms were placed in calculated positions with C—H distances of 0.95 Å (phenyl) and 0.98 Å (methyl), and an O—H distance of 0.84 Å, and with *U*_iso_(phenyl-H) = 1.2*U*_eq_(C), *U*_iso_(methyl-H) = 1.5*U*_eq_(C) and *U*_iso_(hydroxyl-H)=1.5*U*_eq_(O).

### 3. Results and discussion

The ability of aryl­boronic acids to form supra­molecular structures *via* hydrogen-bonding inter­actions of B(OH)_2_ groups is well known. The molecular structure of (I) is shown in Fig. 1. The boronic group is only slightly twisted with respect to the benzene ring whereas the meth­oxy group is twisted almost perpendicularly. The tri­methyl­silyl group is slightly bent with respect to the aromatic ring. The boronic group has an *exo*-*endo* conformation. The *endo*-oriented OH group is engaged into the intra­molecular O—H···O bond with the meth­oxy O atom to form a six-membered ring typical of structures of related *ortho*-alk­oxy­aryl­boronic acids (Cyrański *et al.*, 2012). The molecules of (I) are linked *via* almost linear O—H···O bridges to give centrosymmetric dimers. The periodic calculations performed in PIXEL programme (Gavezzotti, 2003) show that the dimer inter­action energy is equal to -58.5 kJ/mol, which is comparable to the binding energies of other boronic acids dimers reported in the literature (Cyrański *et al.*, 2008; Durka *et al.*, 2012). The supra­molecular architecture in (I) extends through π—π stacking inter­actions of aromatic rings in the parallel-displaced fashion. The boron atoms are also engaged in these mutual inter­actions, which is manifested by a relatively short B1···C2 contact of 3.372 (2) Å. Short B1···C2 inter­actions were described in more detail for the structures of fluorinated 1,4-phenyl­enediboronic acids (Durka *et al.*, 2012). Thus, another centrosymmetric motif can be distinguished. The inter­action energy of such dimers amounts to -33.5 kJ/mol. As a result of H-bonding and π—π inter­actions, a specific columnar network is formed in the *a* axis direction (Figs 2 & 3). The total cohesive energy calculated for asymmetric unit equals to -111.7 kJ/mol. In conclusion, hydrogen-bonding inter­actions of boronic groups are operative to form centrosymmetric dimeric structure of (I). The extended supra­molecular assembly is due to π—π stacking inter­actions of aromatic rings additionally involving the boron atoms.

### Crystal data

**Table:**

C_10_H_17_BO_3_Si	*D*_x_ = 1.218 Mg m^−^^3^
*M**_r_* = 224.14	Melting point: 353 K
Monoclinic, *P*2_1_/*n*	Mo *K*α radiation, λ = 0.71073 Å
*a* = 9.1832 (11) Å	Cell parameters from 1540 reflections
*b* = 9.7082 (10) Å	θ = 2.7–28.7°
*c* = 14.1415 (16) Å	µ = 0.18 mm^−^^1^
β = 104.26 (1)°	*T* = 100 K
*V* = 1221.9 (2) Å^3^	Unshaped, colourless
*Z* = 4	0.16 × 0.12 × 0.10 mm
*F*(000) = 480	

### Data collection

**Table:**

Bruker APEXII diffractometer	2939 independent reflections
Radiation source: TXS rotating anode	2154 reflections with *I* > 2σ(*I*)
Multi-layer optics monochromator	*R*_int_ = 0.029
ω scans	θ_max_ = 28.6°, θ_min_ = 3.0°
Absorption correction: multi-scan (Blessing, 1995)	*h* = −11→10
*T*_min_ = 0.744, *T*_max_ = 0.780	*k* = −13→12
11175 measured reflections	*l* = −18→18

### Refinement

**Table:**

Refinement on *F*^2^	1 restraint
Least-squares matrix: full	Hydrogen site location: inferred from neighbouring sites
*R*[*F*^2^ > 2σ(*F*^2^)] = 0.034	H-atom parameters constrained
*wR*(*F*^2^) = 0.097	*w* = 1/[σ^2^(*F*_o_^2^) + (0.0567*P*)^2^] where *P* = (*F*_o_^2^ + 2*F*_c_^2^)/3
*S* = 1.02	(Δ/σ)_max_ < 0.001
2939 reflections	Δρ_max_ = 0.36 e Å^−^^3^
136 parameters	Δρ_min_ = −0.30 e Å^−^^3^

### Special details

**Table:**

Geometry. All e.s.d.'s (except the e.s.d. in the dihedral angle between two l.s. planes) are estimated using the full covariance matrix. The cell e.s.d.'s are taken into account individually in the estimation of e.s.d.'s in distances, angles and torsion angles; correlations between e.s.d.'s in cell parameters are only used when they are defined by crystal symmetry. An approximate (isotropic) treatment of cell e.s.d.'s is used for estimating e.s.d.'s involving l.s. planes.

### Fractional atomic coordinates and isotropic or equivalent isotropic displacement parameters (Å^2^)

**Table:**

	*x*	*y*	*z*	*U*_iso_*/*U*_eq_	
Si1	0.39406 (5)	0.65611 (4)	0.15682 (3)	0.01408 (12)	
O1	0.70378 (11)	0.73383 (10)	0.12400 (7)	0.0157 (2)	
O3	0.81381 (12)	0.97690 (11)	−0.10209 (7)	0.0183 (2)	
H3	0.9024	1.0067	−0.0879	0.027*	
O2	0.89567 (11)	0.91160 (11)	0.06290 (8)	0.0181 (2)	
H2	0.8636	0.8703	0.1059	0.027*	
C5	0.62375 (16)	0.85346 (14)	−0.02981 (11)	0.0135 (3)	
C1	0.44068 (16)	0.73526 (14)	0.04527 (11)	0.0137 (3)	
C9	0.47875 (18)	0.48146 (15)	0.18637 (11)	0.0178 (3)	
H9A	0.5884	0.4884	0.2002	0.027*	
H9B	0.4423	0.4198	0.1307	0.027*	
H9C	0.4499	0.4447	0.2437	0.027*	
C3	0.35918 (16)	0.84434 (16)	−0.11483 (11)	0.0156 (3)	
H3A	0.2810	0.8643	−0.1710	0.019*	
C6	0.58695 (16)	0.77454 (15)	0.04402 (11)	0.0129 (3)	
C4	0.50551 (17)	0.88657 (15)	−0.11005 (11)	0.0152 (3)	
H4	0.5257	0.9389	−0.1622	0.018*	
C2	0.32783 (16)	0.77273 (15)	−0.03699 (11)	0.0153 (3)	
H2A	0.2267	0.7484	−0.0396	0.018*	
C7	0.77696 (18)	0.60998 (17)	0.10250 (12)	0.0212 (4)	
H7A	0.8574	0.5845	0.1593	0.032*	
H7B	0.8197	0.6265	0.0465	0.032*	
H7C	0.7034	0.5350	0.0871	0.032*	
C8	0.45625 (19)	0.77761 (16)	0.26146 (12)	0.0201 (3)	
H8A	0.4101	0.8680	0.2437	0.030*	
H8B	0.5659	0.7866	0.2773	0.030*	
H8C	0.4254	0.7419	0.3184	0.030*	
C10	0.18585 (17)	0.63586 (17)	0.13142 (13)	0.0212 (4)	
H10A	0.1377	0.7259	0.1156	0.032*	
H10B	0.1592	0.5980	0.1892	0.032*	
H10C	0.1514	0.5731	0.0762	0.032*	
B1	0.78433 (19)	0.91554 (17)	−0.02287 (13)	0.0147 (3)	

### Atomic displacement parameters (Å^2^)

**Table:**

	*U*^11^	*U*^22^	*U*^33^	*U*^12^	*U*^13^	*U*^23^
Si1	0.0142 (2)	0.0130 (2)	0.0156 (2)	−0.00051 (17)	0.00465 (16)	0.00139 (17)
O1	0.0130 (5)	0.0178 (5)	0.0149 (5)	0.0014 (4)	0.0006 (4)	0.0013 (4)
O3	0.0152 (5)	0.0225 (6)	0.0175 (6)	−0.0043 (4)	0.0045 (4)	0.0013 (5)
O2	0.0151 (5)	0.0229 (6)	0.0163 (5)	−0.0054 (5)	0.0036 (4)	0.0018 (5)
C5	0.0145 (7)	0.0115 (7)	0.0148 (7)	−0.0002 (6)	0.0044 (6)	−0.0022 (6)
C1	0.0144 (7)	0.0112 (7)	0.0158 (7)	−0.0001 (6)	0.0045 (6)	−0.0018 (6)
C9	0.0205 (8)	0.0158 (7)	0.0172 (8)	−0.0011 (6)	0.0046 (6)	0.0007 (6)
C3	0.0138 (7)	0.0159 (7)	0.0152 (7)	0.0009 (6)	−0.0001 (6)	0.0010 (6)
C6	0.0129 (7)	0.0120 (7)	0.0127 (7)	0.0009 (6)	0.0010 (6)	−0.0019 (6)
C4	0.0189 (8)	0.0134 (7)	0.0137 (7)	−0.0008 (6)	0.0048 (6)	0.0009 (6)
C2	0.0120 (7)	0.0146 (7)	0.0189 (8)	−0.0007 (6)	0.0032 (6)	−0.0018 (6)
C7	0.0189 (8)	0.0213 (8)	0.0235 (9)	0.0057 (6)	0.0054 (7)	0.0040 (7)
C8	0.0259 (9)	0.0175 (8)	0.0182 (8)	−0.0007 (7)	0.0080 (7)	0.0000 (7)
C10	0.0174 (8)	0.0217 (8)	0.0262 (9)	−0.0011 (6)	0.0084 (7)	0.0035 (7)
B1	0.0154 (8)	0.0128 (8)	0.0168 (9)	0.0006 (7)	0.0055 (7)	−0.0025 (7)

### Geometric parameters (Å, º)

**Table:**

Si1—C10	1.8668 (16)	C9—H9B	0.9800
Si1—C8	1.8684 (16)	C9—H9C	0.9800
Si1—C9	1.8693 (16)	C3—C2	1.391 (2)
Si1—C1	1.8960 (15)	C3—C4	1.390 (2)
O1—C6	1.4103 (17)	C3—H3A	0.9500
O1—C7	1.4458 (18)	C4—H4	0.9500
O3—B1	1.354 (2)	C2—H2A	0.9500
O3—H3	0.8400	C7—H7A	0.9800
O2—B1	1.381 (2)	C7—H7B	0.9800
O2—H2	0.8400	C7—H7C	0.9800
C5—C6	1.402 (2)	C8—H8A	0.9800
C5—C4	1.401 (2)	C8—H8B	0.9800
C5—B1	1.574 (2)	C8—H8C	0.9800
C1—C2	1.402 (2)	C10—H10A	0.9800
C1—C6	1.401 (2)	C10—H10B	0.9800
C9—H9A	0.9800	C10—H10C	0.9800
			
C10—Si1—C8	108.52 (8)	C3—C4—C5	121.24 (14)
C10—Si1—C9	107.31 (7)	C3—C4—H4	119.4
C8—Si1—C9	111.49 (7)	C5—C4—H4	119.4
C10—Si1—C1	108.24 (7)	C3—C2—C1	122.12 (14)
C8—Si1—C1	108.39 (7)	C3—C2—H2A	118.9
C9—Si1—C1	112.76 (7)	C1—C2—H2A	118.9
C6—O1—C7	111.44 (11)	O1—C7—H7A	109.5
B1—O3—H3	109.5	O1—C7—H7B	109.5
B1—O2—H2	109.5	H7A—C7—H7B	109.5
C6—C5—C4	116.59 (13)	O1—C7—H7C	109.5
C6—C5—B1	123.85 (13)	H7A—C7—H7C	109.5
C4—C5—B1	119.35 (13)	H7B—C7—H7C	109.5
C2—C1—C6	115.73 (13)	Si1—C8—H8A	109.5
C2—C1—Si1	121.55 (11)	Si1—C8—H8B	109.5
C6—C1—Si1	122.32 (11)	H8A—C8—H8B	109.5
Si1—C9—H9A	109.5	Si1—C8—H8C	109.5
Si1—C9—H9B	109.5	H8A—C8—H8C	109.5
H9A—C9—H9B	109.5	H8B—C8—H8C	109.5
Si1—C9—H9C	109.5	Si1—C10—H10A	109.5
H9A—C9—H9C	109.5	Si1—C10—H10B	109.5
H9B—C9—H9C	109.5	H10A—C10—H10B	109.5
C2—C3—C4	119.67 (14)	Si1—C10—H10C	109.5
C2—C3—H3A	120.2	H10A—C10—H10C	109.5
C4—C3—H3A	120.2	H10B—C10—H10C	109.5
C5—C6—C1	124.48 (14)	O3—B1—O2	118.90 (14)
C5—C6—O1	118.37 (12)	O3—B1—C5	119.49 (14)
C1—C6—O1	117.14 (13)	O2—B1—C5	121.60 (14)
			
C10—Si1—C1—C2	2.07 (14)	Si1—C1—C6—O1	9.56 (18)
C8—Si1—C1—C2	−115.45 (13)	C7—O1—C6—C5	−83.99 (16)
C9—Si1—C1—C2	120.62 (12)	C7—O1—C6—C1	97.38 (15)
C10—Si1—C1—C6	174.45 (12)	C2—C3—C4—C5	2.7 (2)
C8—Si1—C1—C6	56.93 (14)	C6—C5—C4—C3	0.8 (2)
C9—Si1—C1—C6	−67.00 (14)	B1—C5—C4—C3	−174.09 (14)
C4—C5—C6—C1	−4.2 (2)	C4—C3—C2—C1	−3.1 (2)
B1—C5—C6—C1	170.43 (14)	C6—C1—C2—C3	0.0 (2)
C4—C5—C6—O1	177.24 (12)	Si1—C1—C2—C3	172.85 (11)
B1—C5—C6—O1	−8.1 (2)	C6—C5—B1—O3	172.48 (14)
C2—C1—C6—C5	3.8 (2)	C4—C5—B1—O3	−13.0 (2)
Si1—C1—C6—C5	−168.98 (11)	C6—C5—B1—O2	−8.9 (2)
C2—C1—C6—O1	−177.64 (12)	C4—C5—B1—O2	165.67 (14)

### Hydrogen-bond geometry (Å, º)

**Table:**

*D*—H···*A*	*D*—H	H···*A*	*D*···*A*	*D*—H···*A*
O2—H2···O1	0.84	2.04	2.7532 (14)	142
O3—H3···O2^i^	0.84	1.97	2.8051 (14)	175

Symmetry code: (i) −*x*+2, −*y*+2, −*z*.
